# Class B Gene Expression and the Modified ABC Model in Nongrass Monocots

**DOI:** 10.1100/tsw.2007.86

**Published:** 2007-02-19

**Authors:** Akira Kanno, Mutsumi Nakada, Yusuke Akita, Masayo Hirai

**Affiliations:** Graduate School of Life Sciences, Tohoku University, Katahira 2-1-1, Aoba-ku, Sendai 980-8577, Japan

**Keywords:** modified ABC model, class B gene, tulip, lily, Agapanthus praecox, Muscari armeniacum, Tricyrtis affinis, asparagus, Phalaenopsis equestris, Crocus sativus, Dendrobium crumenatum

## Abstract

The discovery of the MADS-box genes and the study of model plants such as Arabidopsis thaliana and Antirrhinum majus have greatly improved our understanding of the molecular mechanisms driving the diversity in floral development. The class B genes, which belong to the MADS-box gene family, are important regulators of the development of petals and stamens in flowering plants. Many nongrass monocot flowers have two whorls of petaloid organs, which are called tepals. To explain this floral morphology, the modified ABC model was proposed. This model was exemplified by the tulip, in which expansion and restriction of class B gene expression is linked to the transition of floral morphologies in whorl 1. The expression patterns of class B genes from many monocot species nicely fit this model; however, those from some species, such as asparagus, do not. In this review, we summarize the relationship between class B gene expression and floral morphology in nongrass monocots, such as Liliales (Liliaceae) and Asparagales species, and discuss the applicability of the modified ABC model to monocot flowers.

## INTRODUCTION

Flower development in plants can be subdivided into several major steps, including floral induction, floral meristem formation, and floral organ development. Genetic control of the different steps of flower development is achieved by a hierarchy of interacting regulatory genes[1]. The identification of floral organ development as one of the steps in flower development was recognized during studies of homeotic mutants in which the identity of floral organs was changed. Genetic mutants of *Arabidopsis thaliana* and *Antirrhinum majus* were categorized into three classes (A, B, and C), and functional analyses of these mutants led to the ABC model of flower development[2]. Class A mutants have carpels in the first whorl instead of sepals, and stamens in the second whorl instead of petals. Class B mutants have sepals rather than petals in the second whorl and carpels rather than stamens in the third whorl. Class C mutants have petals instead of stamens in the third whorl and sepals instead of carpels in the fourth whorl, and an additional flower arises in the fourth whorl. Based on these classes of single mutants, and all combinations of double and triple mutants, the ABC model proposed that there are three functional classes of floral organ identity genes, called A, B, and C. The activity of class A genes (*APETALA1* [*AP1*]

and *APETALA2* [*AP2*] in *A. thaliana*, *SQUAMOSA* in *A. majus*) is required in the outer two whorls (first and second), class B genes (*APETALA3* [*AP3*] and *PISTILLATA* [*PI*] in *A. thaliana*, *DEFICIENS* [*DEF*] and *GLOBOSA* [*GLO*] in *A. majus*) function in the second and third whorls, and class C genes (*AGAMOUS* [*AG*] in *A. thaliana*, *PLENA* in *A. majus*) act in the inner two whorls (third and fourth). More recently, this model was extended to include class D genes, which specify the identity of ovules within the carpel[1,3,4]. An additional group of floral organ identity genes, the E-class genes (*SEPALLATA* [*SEP*],[5]), are involved in specifying petals, stamens, and carpels[6,7]. The model has therefore been renamed the ABCDE model of flower development. All genes involved in the ABCDE model, except for *AP2*, belong to the family of MADS-box genes that encode MADS-box transcription factors[8].

## Modified ABC Model in Tulip

In contrast to the flowers of higher eudicots, many nongrass monocot flowers, such as lily and tulip, have three outer tepals, three inner tepals, six stamens, and three carpels (Fig. 1A). It is difficult to fully account for this type of floral morphology using the classical ABC model. To explain this floral morphology, van Tunen *et al*. proposed a modified ABC model: the expression of class B genes was extended to whorl 1, with the result that the floral organs of the first and second whorls have almost the same petaloid structure (Fig. 1B, [9]). This model was based on the morphology of wild-type and mutant flowers of tulip (*Tulipa gesneriana*, Liliaceae). In a *viridiflora* tulip mutant, the organs of the outer two whorls are greenish and resemble the sepals of higher eudicots, and the six stamens are transformed into carpeloid organs. Another mutant flower has tepal-like structures in whorls 1, 2, and 3, and a new floral structure arises from the center of the flower. These phenotypes are similar to the phenotypes observed for class B and class C mutants of higher eudicots, respectively. Based on these morphological observations, van Tunen *et al*. proposed the modified ABC model, although they did not analyze these mutants using genetics or molecular biology.

Ten years after the modified ABC model was proposed, putative class B genes were isolated from tulip[10], including two *DEF*-like genes (*TGDEFA*, *TGDEFB*) and one *GLO*-like gene (*TGGLO*). The transcripts of all three genes are detected in floral organs and not in vegetative organs. *TGDEFA* and *TGDEFB* are strongly expressed in whorls 1, 2, and 3, and *TGGLO* is strongly expressed in the outer three whorls and weakly in carpels (Fig. 1C). In *A. thaliana* and *A. majus*, both types of class B genes (*DEF*- and *GLO*-like genes) are needed for B function because their gene products function as heterodimers, and both *DEF*-like and *GLO*-like genes are expressed in outer and inner tepals and stamens in tulip. This expression pattern nicely fits the modified ABC model.

## The Expression of Class B Genes in Other Monocots

### *Liliaceae (Liliales)*

Several class B genes were isolated from lily, a liliaceous plant closely related to tulip. One *DEF*-like (*LRDEF*) and two *GLO*-like (*LRGLOA* and *LRGLOB*) genes has been isolated from *Lilium regale* (Fig. 2,[1,11]), and one *DEF*-like gene (*LMADS1*) has been isolated from *L. longiflorum*[12]. A Northern blot analysis of dissected floral organs showed that the *LRDEF* gene is expressed in outer and inner tepals and stamens. This expression pattern, like that of tulip, supports the modified ABC model [1]. On the other hand, the expression pattern of *LMADS1* is slightly different from that of *LRDEF*. A Northern blot analysis showed that *LMADS1* is expressed strongly in whorls 2 and 3 and weakly in whorls 1 and 4, whereas its protein accumulates only in whorls 2 and 3[12]. These results suggest the possibility that the floral morphology of *L. longiflorum*, which has two whorls of petaloid tepals, cannot be explained by the

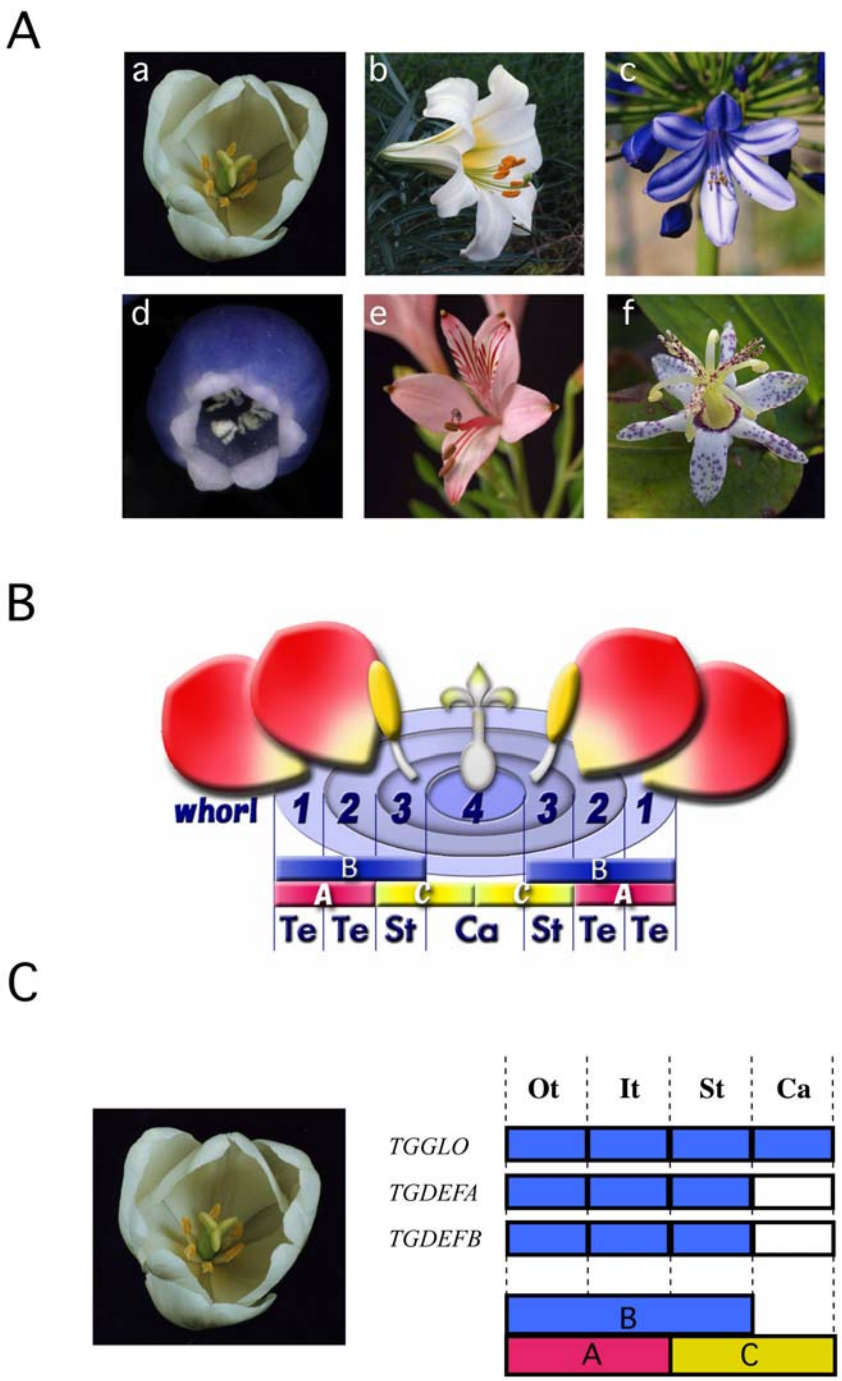

**Figure 1**. A. Monocot flowers with two whorls of petaloid organs. *Tulipa gesneriana* (a), *Lilium regale* (b), *Agapanthus praecox* (c), *Muscari armeniacum* (d), *Alstroemeria ligtu* (e), and *Tricyrtis affinis* (f). B. Modified ABC model proposed by van Tunen *et al*.[9]. This model explains the flower morphology of tulip: class B genes are expressed in whorl 1 as well as whorls 2 and 3, thus the organs of whorl 1 have the same petaloid character as those of whorl 2. C. The expression pattern of the class B genes (*TGGLO*, *TGDEFA*, and *TGDEFB*) from *Tulipa gesneriana*. Te, tepals; Ot, outer tepals; It, inner tepals; St, stamens; Ca, carpels.

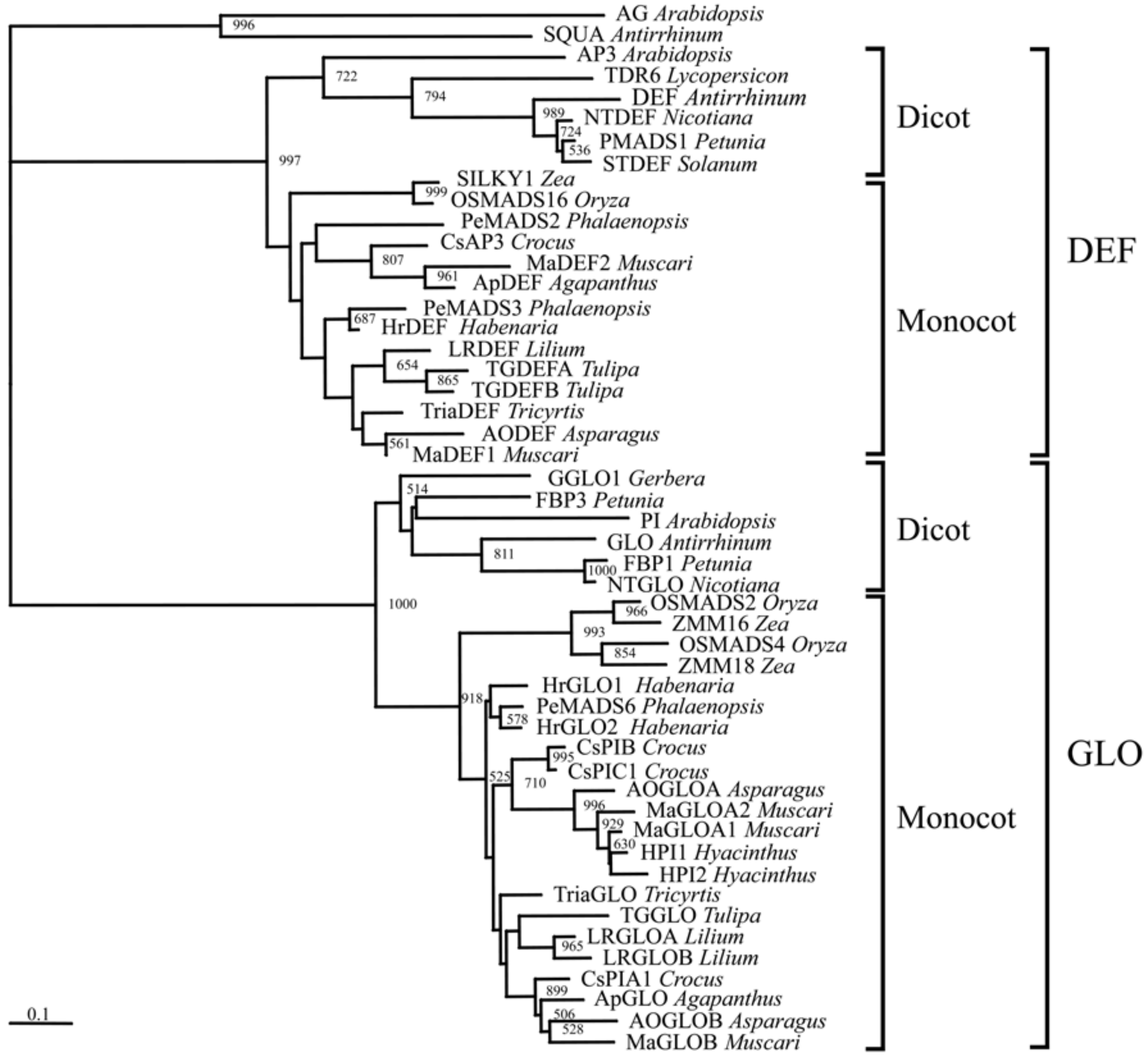

**Figure 2.** Phylogenetic tree of *DEF*- and *GLO*-like genes. The neighbor-joining method was used. The numbers next to the nodes give bootstrap values from 1,000 replicates.

modified ABC model. However, these expression analyses with Northern hybridization and Western blotting were performed using dissected floral organs. In order to clarify these inconsistent results in the two lily species, the accumulation of the mRNA and protein of class B genes should be analyzed in floral organ primordia using flowers at very early stages. Given that multiple *DEF*-like genes exist in tulip, the possibility also remains that another lily *DEF*-like gene is expressed in the outer whorl of tepals.

A better functional understanding of *DEF*- and *GLO*-like genes in Liliaceae plants would be obtained by transgenic approach, but the genetic transformation of lily and tulip is very difficult. Therefore, we have started to isolate class B genes from another Liliaceae plant, *Tricyrtis affinis*, because *Agrobacterium*-mediated transformation system has been recently developed in *Tricyrtis* species[13]. We have already isolated *TriaDEF* and *TriaGLO* genes from *T. affinis* (Fig. 2). An RT-PCR analysis showed that *TriaDEF* is expressed in whorls 1, 2, and 3, whereas *TriaGLO* is expressed in all floral organs (Fig. 3A: our unpublished result). These results in *T. affinis* are consistent with a modified ABC model.

## Asparagales

Like Liliaceae plants, most flowers in Asparagales have two whorls of petaloid tepals. Many class B genes have been isolated from several plants belonging to Asparagales, and their gene expression patterns have been analyzed. Most of the results support the modified ABC model.

*Agapanthus praecox*, which is a member of Asparagales, has two whorls of almost identical petaloid organs (Fig. 1A, panel c). *ApDEF* and *ApGLO* have been isolated from this plant (Fig. 2, [14]). An RT-PCR analysis detected *ApGLO* expression in whorls 1, 2, and 3, whereas *ApDEF* transcripts were detected in all floral organs (Fig. 3B,[14]). The expression patterns of these genes were also analyzed by *in situ* hybridization, which showed that both of these genes are expressed in whorl 1 as well as in whorls 2 and 3 during the early stages of floral development in *A. praecox* (Fig. 3B:[14]). This is the first report to support the modified ABC model and to show that the class B genes are expressed in the floral organ primordia of the two outer whorls.

*Muscari armeniacum*, another member of Asparagales, has two whorls of petaloid tepals fused into a tube (Fig. 1A, panel d). One *DEF*-like gene, *MaDEF1* (formerly *MaDEF*), has been isolated from this plant species[15]. An RT-PCR analysis using total RNA isolated from dissected floral organs by the laser microdissection system detected the *MaDEF1* transcript in whorls 1, 2, and 3[15]. Later, an additional *DEF*-like gene (*MaDEF2*) and three *GLO*-like genes (*MaGLOA1*, *MaGLOA2* and *MaGLOB*) were isolated from *M. armeniacum* (Fig. 2: our unpublished result). An RT-PCR analysis detected the transcripts of *MaDEF2* and the three *GLO*-like genes in all floral organs (Fig. 4A: our unpublished result). The extended expression of class B genes in whorl 1 fits the modified ABC model. The additional expression of class B genes in whorl 4, which is not consistent with this model, is discussed in next section, “Unsolved problems of the modified ABC model.”

Recently, *DEF*-like (*CsatAP3*) and *GLO*-like (*CsatPIc*) genes were isolated from *Crocus sativus*[16]. An RT-PCR analysis detected the transcripts of both of these genes in all floral organs. This result of expanded class B gene expression in whorl 1 is similar to that found in *M. armeniacum*, supporting the modified ABC model.

The orchids (Orchidaceae) are a huge family within the order Asparagales whose species are distributed throughout the world and vary greatly in morphology. So far, class B genes have been isolated from three orchid species, *Phalaenopsis equestris*[17,18], *Dendrobium crumenatum* [19], and *Oncidium* Gower Ramsey[20], although no *GLO*-like gene of *Oncidium* has been reported. Four *DEF*-like genes, *PeMADS2*, *PeMADS3, PeMADS4*, and *PeMADS5*[17], and one *GLO*-like gene, *PeMADS6*[18], have been reported in *P. equestris*, one of the most important horticultural plants among orchids. The four *DEF*-like genes are differentially expressed. The transcripts of *PeMADS2* and *PeMADS5* are found in petaloid sepals, petals (including lips), and columns, but are not found in pollinia. *PeMADS3* is expressed in petals, lips, and columns, whereas the expression of *PeMADS4* is restricted to lips and columns. *PeMADS6* transcripts are detected in *P. equestris* in the whorl 1 sepals and whorl 2 petals, as well as lips and columns (Fig. 3C). The expanded expression of *DEF*-like (*PeMADS2* and *PeMADS5*) and *GLO*-like (*PeMADS6*) genes in whorl 1 supports the modified ABC model.

Several MADS-box genes containing class B genes were reported in another orchid, *D. crumenatum*[19]. From *D. crumenatum*, two *DEF*-like genes (*DcOAP3A* and *DcOAP3B*) and one *GLO*-like gene (*DcOPI*) were isolated and the localization of their mRNA was examined. *DcOAP3A* mRNA was detected in all floral organs and leaves, whereas *DcOAP3B* mRNA was found in petals and lips (whorl 2), anthers (whorl 3), and columns (whorl 4, excluded anthers and their caps). In contrast, *DcOPI* was expressed in all floral organs. These expression patterns were also confirmed by *in situ* hybridization. These results in *D. crumenatum* are reminiscent of those in *C. sativus* and *M. armeniacum*.

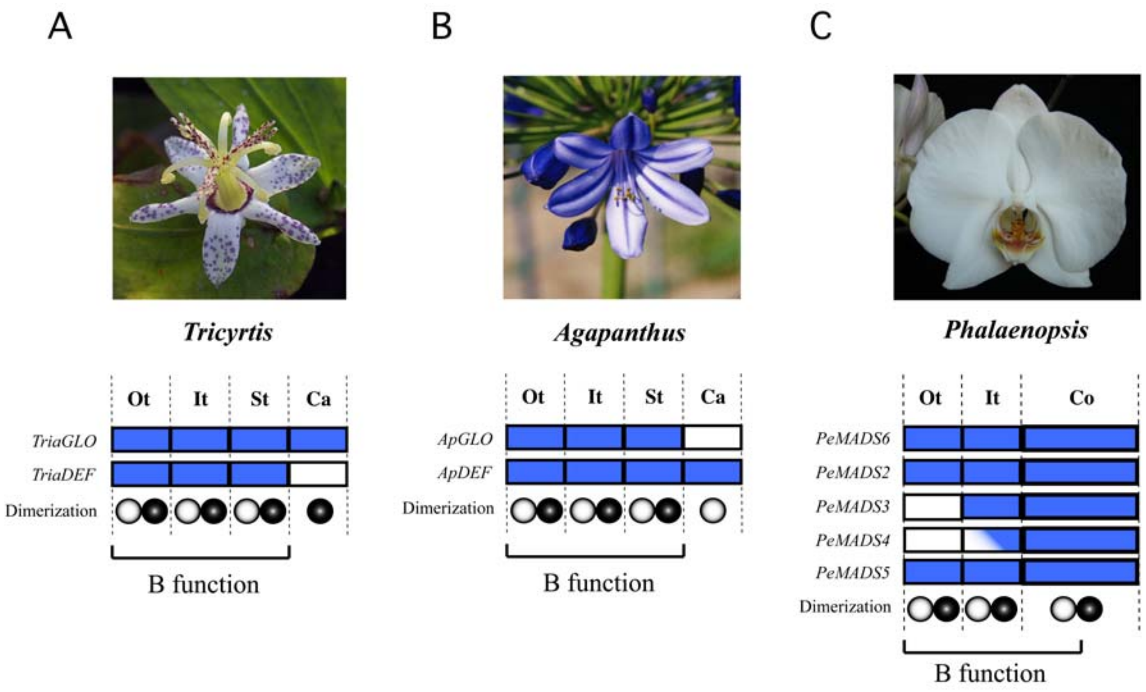

**Figure 3.** The expression pattern of the class B genes from *Tricyrtis affinis* (A), *Agapanthus praecox* (B), and *Phalaenopsis equestris* (C). RT-PCR and/or Northern hybridization showing the organ-specific expression in each whorl. Shaded boxes indicate the expression of class B genes. The expression of *P. equestris PeMADS4* is only observed in the lip and not in other inner tepals. These expression patterns of the class B genes support a modified ABC model. The dimerization pattern is shown below the expression patterns. Open and closed circles show DEF-like and GLO-like proteins, respectively. Ot, outer tepals; It, inner tepals; St, stamens; Ca, carpels; Co, columns.

Phylogenetic analysis showed that monophyly of monocot *GLO*-like genes is well supported (Fig. 2). It is interesting to note that the *GLO*-like genes from Asparagales are divided into two clusters in the phylogenetic tree: one cluster contains *CsPIA1* (*C. sativus*), *ApGLO* (*A. praecox*), *AOGLOB* (*Asparagus officinalis*), and *MaGLOB* (*M. armeniacum*), and the other group contains *CsPIB* and *CsPIC1* (*C. sativus*), *AOGLOA* (*A. officinalis*), *MaGLOA1* and *MaGLOA2* (*M. armeniacum*), and *HPI1* and *HPI2* (*Hyacinthus orientalis*). This indicates that both recent and ancient gene duplication events occurred during the evolution of Asparagales, and the ancient duplication that generated the *GLO*-like genes in these two clusters occurred before the divergence of these genera. *DEF*-like genes from Asparagales are also divided into two clusters: one includes *MaDEF1* (*M. armeniacum*) and *AODEF* (*A. officinalis*), and the other contains *MaDEF2* (*M. armeniacum*), *ApDEF* (*A. praecox*), and *CsAP3* (*C. sativus*). This indicates that, as with the *GLO*-like genes, the gene duplication event in *DEF*-like genes occurred before the divergence of these genera. *DEF*-like genes from *M. armeniacum* fall into each cluster, but it is not clear whether two (or more) *DEF*-like genes might exist in other plants in Asparagales, such as *C. sativus* and *A. praecox*.

## Unsolved Problems of The Modified ABC Model

Many of the data on class B gene expression support the modified ABC model in Liliales (Liliaceae) and Asparagales, showing that petaloid tepals in monocots might correlate with the expansion of class B gene expression. However, there are some interesting results in monocot species that are not easy to explain.

### *I. DEF-* and *GLO-Like Genes Are Not Expressed in Whorl 1 in Garden Asparagus*

Garden asparagus (*Asparagus officinalis*) has two whorls of almost identical petaloid tepals, similar to lily and tulip. One *DEF*-like (*AODEF*) and two *GLO*-like (*AOGLOA*, *AOGLOB*) genes have been isolated from asparagus, and *in situ* hybridization showed that the expression of these genes is restricted to whorls 2 and 3 (inner tepals and stamens, respectively); no transcripts are detected in whorl 1 (outer tepals; Fig. 4B)[21,22]. Since both *DEF*- and *GLO*-like genes are required for the B-function in eudicots, it is likely that the B-function in asparagus is restricted to whorls 2 and 3. Thus, the results of the asparagus class B gene expression do not support the modified ABC model, which proposes that two whorls of petaloid tepals are formed due to expanded class B gene expression in addition to class A gene expression. Is there no B-function in whorl 1 in asparagus? There are three hypotheses explaining the floral morphology in asparagus: (1) these class B genes are not involved in tepal development, (2) there are additional *DEF*- and *GLO*-like class B genes that are specifically involved in outer tepal development, and (3) class B genes are transcribed in whorls 2 and 3 and the products move to whorl 1 (see[23]). In asparagus, one floral homeotic mutant is known in which the stamens are homeotically changed to carpels and the petaloid tepals are changed to sepaloid or leaf-like structures[23,24]. Because the mutant phenotype is very similar to that of the tulip "B mutant" reported previously[9], we favor the second hypothesis. There might be several class B genes in asparagus, but only three have already been isolated, and the regulatory mechanism of the expression of these class B genes may be damaged in the mutant; thus, the phenotype may arise from the lack of B function in whorls 1, 2, and 3. Further genetic and molecular biological analyses using this mutant are needed to clarify the mechanism of floral development in asparagus.

### *II. Both* DEF- *and* GLO-*Like Genes Are Expressed in Whorl 4 in* Dendrobium crumenatum, Crocus sativus, *and* Muscari armeniacum

In the ABC model, B-function genes work with A-function genes to specify the development of petals and with C-function genes to specify the development of stamens. In *A. thaliana*, transcripts of *PI* are detectable in whorls 2, 3, and 4, whereas those of *AP3* are found in whorls 1, 2, and 3[25,26]. In tobacco, *GLO*-like gene (*NTGLO*) transcripts are restricted to the second and third whorls, and *DEF*-like gene (*NTDEF*) transcripts are detectable in all floral organs[27]. This class B gene expression in tobacco is similar to that in *A. majus*[8]. The expression of *DEF*- and *GLO*-like genes overlaps in the second and third whorls and their gene products perform B functions in these plants. To date, many *DEF*- and *GLO*-like genes have been isolated from various plant species. Expression studies (summarized in[28]) have shown that the overlapping expression of *DEF*- and *GLO*-like genes is restricted to stamens and petals (including petaloid tepals). In monocots with two whorls of petaloid tepals, overlapping expression of *DEF*- and *GLO*-like genes is found in outer and inner tepals and stamens in tulip (Fig. 1C), *A. praecox*, and *T. affinis* (Fig. 3A, B). In contrast to these results, the additional expression of both *DEF*- and *GLO*-like genes in whorl 4 has been reported in 2 species.

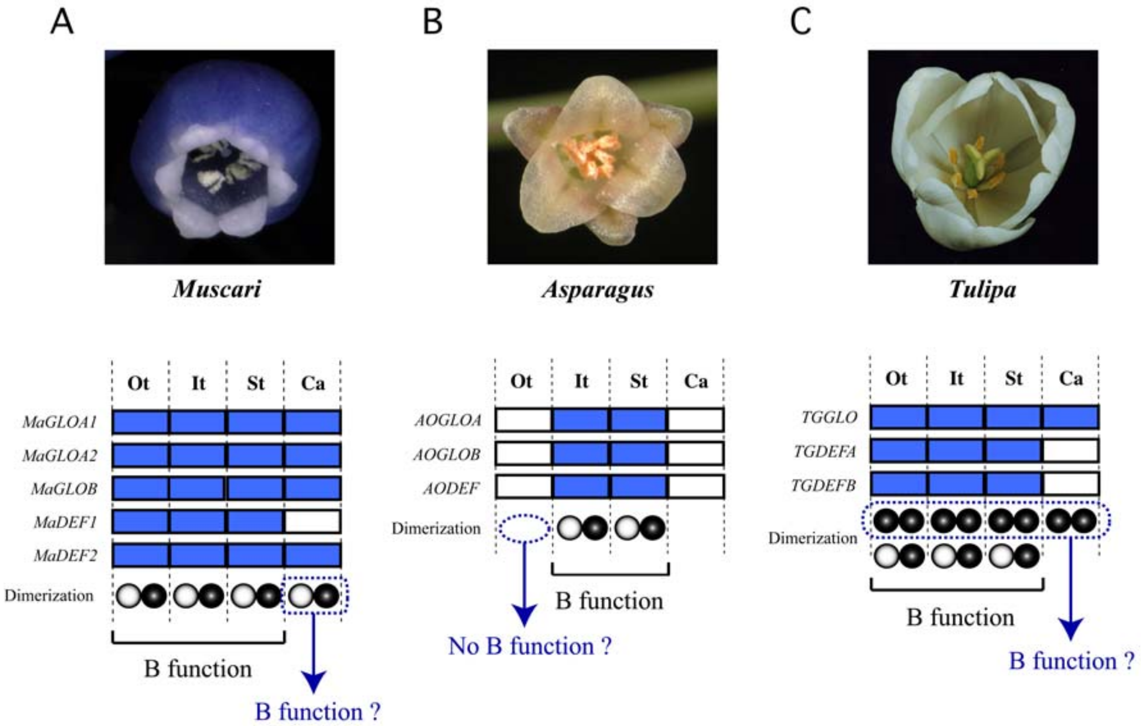

**Figure 4.** The expression pattern of the class B genes from *Muscari armeniacum* (A), *Asparagus officinalis* (B), and *Tulipa gesneriana* (C). Shaded boxes indicate the expression of class B genes. The expression patterns of some of these genes indicate that the modified ABC model is not strictly applicable to these monocots. The dimerization patterns are shown below the expression patterns. Open and closed circles show DEF-like and GLO-like proteins, respectively. Ot, outer tepals; It, inner tepals; St, stamens; Ca, carpels.

Recently, three B-function genes, *DcOAP3A*, *DcOAP3B*, and *DcOPI*, were isolated from the orchid *D. crumenatum*[19]. *DcOAP3A* and *DcOPI* are expressed in all floral organs, including petaloid sepals, petals, lips, and male and female reproductive organs, whereas *DcOAP3B* is expressed in the second whorl of petals and lips and in the reproductive organs including pollinia and columns. All three genes, *DcOAP3A*, *DcOAP3B*, and *DcOPI*, are expressed in the lower part of the column, which is equivalent to whorl 4 (carpels) in *A. thaliana*. This expanded expression of class B genes in whorl 4 was also detected in crocus[16]. An RT-PCR expression analysis indicated that *CsatAP3* and *CsatPIc* (*DEF*- and *GLO*-like genes, respectively, from crocus) are expressed in all of four floral organs. Moreover, an RT-PCR analysis in *M. armeniacum* detected the transcripts of three *GLO*-like genes (*MaGLOA1*, *MaGLOA2*, and *MaGLOB*) in all floral organs, and one *DEF*-like gene (*MaDEF2*) was expressed in whorl 4 as well as whorls 1, 2, and 3 (Fig. 4A: our unpublished result). The extended expression of class B genes into whorl 1 fits the modified ABC model; however, the additional expression in whorl 4 is not consistent with this model. Is there B-function in whorl 4 in these plants? There are two possibilities to explain this result. One possibility is that *DEF*- and *GLO*-like gene expression is regulated post-transcriptionally, and the proteins are not synthesized in whorl 4. In *L. longiflorum*, the mRNA of a *DEF*-like gene (*LMADS1*) was detected strongly in whorls 2 and 3, and also weakly in whorls 1 and 4, whereas the LMADS1 protein was only detected in whorls 2 and 3, suggesting that the expression of *LMADS1* is regulated post-transcriptionally[12]. Thus, it would be very interesting to analyze protein expression in whorl 4 in *D. crumenatum*, *C. sativus*, and *M. armeniacum*. Recent publications indicate that microRNAs can play regulatory roles in animals and plants by targeting mRNAs for cleavage or translational repression[29]. Therefore, microRNAs may act as negative regulators of class B gene expression, although direct regulation of MADS-box genes by microRNAs has not been reported yet. The other possibility is that the

DEF/GLO-heterodimer of these species needs additional factor(s), which are not expressed in whorl 4, to have B-function in these plants. Based on the floral quartet model [30], the AP3/PI heterodimer has multimeric protein interactions with AP1, SEP, and AG in *A. thaliana*. In *D. crumenatum*, yeast two-hybrid analyses have shown that the DEF-like proteins DcOAP3A and DcOAP3B form heterodimers with a GLO-like protein, DcOPI. Moreover, these DEF/GLO heterodimers interact with a SEP-like protein, DcOSEP, to form higher protein complexes. The transcripts of these genes, including *DcOSEP*, were detected in all floral organs, indicating that these products could form multimeric complexes in all four whorls. If there are factors that interact with *D. crumenatum* DEF/GLO-heterodimer, they would likely be MADS-box proteins like DcOSEP, although there is no evidence of these additional factors.

### *III. Homodimerization of GLO-Like Proteins in Lily and Tulip*

In *A. thaliana* and *A. majus*, B function is provided by heterodimers of DEF- and GLO-like proteins. In tulip, gel retardation assays showed that two DEF-like proteins (TGDEFA and TGDEFB) cannot homodimerize, but instead form heterodimers with GLO-like proteins (TGGLO), as do eudicot DEF- and GLO-like proteins (Fig. 4C,[10]). The *TGGLO* gene in tulip is expressed in all floral organs, including carpels. Two *DEF*-like genes are expressed together with the *TGGLO* gene in whorls 1, 2, and 3, so that the DEF/GLO heterodimer would provide B function in these organs. However, it is very interesting that TGGLO can bind to the CArG-box transcription factor binding site as a homodimer. Therefore, TGGLO homodimer would exist in whorl 4 in tulip. Homodimerization of GLO-like proteins have also been reported in a lily, *Lilium regale* (LRGLOA and LRGLOB,[11]), although the expression of lily *GLO*-like genes has not been analyzed. Do these homodimers have B function or another function that is not known in *A. thaliana* or *A. majus*? LMADS1 (a DEF-like protein from *L. longiflorum*) can also form a homodimer, which was identified in a yeast two-hybrid assay[12]. Although the expression of LMADS1 is restricted to whorls 2 and 3, the function of the LMADS1 homodimer also should be clarified.

## SUMMARY AND PERSPECTIVES

Many monocot flowers have petaloid perianths in whorls 1 and 2, and it is difficult to fully account for this type of floral morphology using the classical ABC model. On the basis of morphological analyses of tulip mutants, van Tunen *et al.*[9] hypothesized that the formation of tepals is due to the expanded expression of class B genes into whorl 1. Moreover, a number of studies in nongrass monocots, such as tulip[10], *P. equestris*[17, 18], *A. praecox*[14], *M. armeniacum*[15], and *D. crumenatum*[19], lend support to a simple modification of the ABC model, the so-called modified ABC model[9]. In addition to being expressed in monocots, the AP3 and PI class B gene homologues are expressed in petaloid sepals in the first whorl in two dicots, *Aquilegia alpina* and *A. clematiflora* of Ranunculaceae[31]. The expansion of class B gene expression into the first whorl is consistent with the floral morphology of these species. Considering that the data are similar in monocots and dicots, the morphologies of whorls 1 and 2 may be generally linked to the expanded expression of class B genes. However, this does not apply in garden asparagus. In asparagus, the expression of class B genes is restricted to whorls 2 and 3, despite the presence of almost identical petaloid perianths in whorls 1 and 2[21,22]. In *D. crumenatum*, *C. sativus*, and *M. armeniacum*, the class B genes are expressed in whorls 1, 2, and 3, which fits the modified ABC model, but are also expressed in whorl 4, which does not fit the modified ABC model. However, the protein localization of the class B gene products is still unclear.

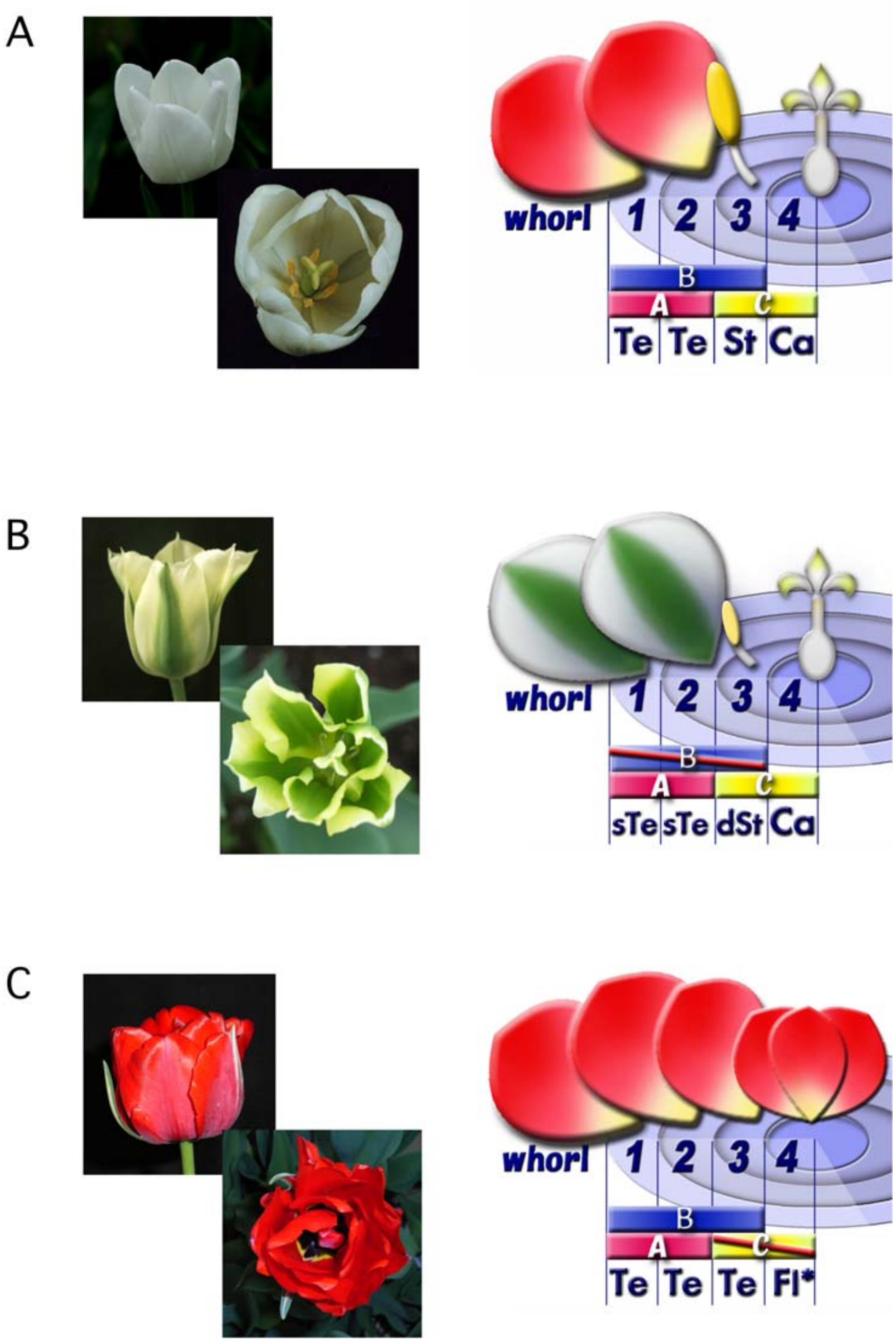

**Figure 5.** Various tulip flowers (left) and the models explaining these flower morphologies (right). A. The wild-type tulip flower has two whorls of petaloid tepals, stamens, and carpels. B. The *viridiflora* tulip flower has two whorls of partially greenish tepals (sepaloid), degenerated stamens, and carpels. This floral morphology is explained by reduced expression of class B gene(s). C. The double-flowered tulip has petaloid tepals in whorl 3 in addition to the outer two whorls and a new flower in the innermost whorl. This floral morphology is explained by reduced expression of the class C gene in whorls 3 and 4. Te, tepals; St, stamens; Ca, carpels; sTe, sepaloid tepals; dSt, degenerated stamens; Fl*, new flower.

In order to clarify the molecular mechanism of petaloid tepal development in nongrass monocots, such as Liliales and Asparagales, functional studies with mutant analyses and genetic transformation are needed. Many of the species mentioned in this review are ornamental plants, and some known ornamental varieties are shown in figure 5. The wild-type tulip has two outer whorls of almost identical petaloid organs (Fig. 5A), whereas the viridiflora tulip has greenish ('sepaloid') tepals in whorls 1 and 2 and slightly degenerated stamens in whorl 3 that can be explained in the modified ABC model by a loss of B function (Fig. 5B). The double-flowered tulip has petaloid tepals in whorl 3 and the innermost whorl has become a new flower, which can be explained by a loss of C function (Fig. 5C). The genetic analysis of these tulip mutants is difficult because it takes several years to get seeds of next generation, and the expression patterns of floral homeotic genes in these mutants are therefore very helpful for clarifying the mechanism of floral organ development in these plants. Recently, a system for producing transgenic plants has been developed for *M. armeniacum*, *A. praecox*, and *Tricyrtis hirta* via *Agrobacterium*-mediated transformation[13,32,33]. Functional studies using this transformation technique will help illuminate the mechanisms of petaloid organ differentiation in monocots with two whorls of petaloid tepals.

## ACKNOWLEDGMENTS

We deeply thank Dr. Masaru Nakano (Niigata University, Japan), Dr. Yoichiro Hoshino (Hokkaido University, Japan), and all of our lab members for helpful comments and discussion. This work was partly supported by a Grant-in-Aid for Scientific Research from the Ministry of Education, Culture, Sports, Science, and Technology of Japan.
